# Anticipated Notification of Sexual Partners following STD Diagnosis among Men Who Have Sex with Men and Transgender Women in Lima, Peru: A Mixed Methods Analysis

**DOI:** 10.1371/journal.pone.0163905

**Published:** 2016-09-29

**Authors:** Jesse L. Clark, Amaya G. Perez-Brumer, Eddy R. Segura, Hector J. Salvatierra, Jorge Sanchez, Javier R. Lama

**Affiliations:** 1 UCLA Geffen School of Medicine, Department of Medicine, Division of Infectious Diseases, Los Angeles, CA, United States of America; 2 Columbia University Mailman School of Public Health, New York, NY, United States of America; 3 Asociacion Civil Impacta Salud y Educacion, Lima, Peru; University of New South Wales, AUSTRALIA

## Abstract

**Background:**

New strategies to support partner notification (PN) are critical for STD control and require detailed understanding of how specific individual and partnership characteristics guide notification decisions.

**Methods:**

From 2011 to 2012, 397 MSM and TW recently diagnosed with HIV, syphilis, or another STD completed a survey on anticipated notification of recent sexual partners and associated factors. Qualitative interviews were conducted with a subset of participants to provide further depth to quantitative findings. Prevalence ratios and generalized estimating equation (GEE) models were used to analyze participant- and partner-level factors associated with anticipated PN.

**Results:**

Among all partners reported, 52.5% were described as “Very Likely” or “Somewhat Likely” to be notified. Anticipated notification was more likely for main partners than casual (adjusted Prevalence Ratio [aPR], 95% CI: 0.63, 0.54–0.75) or commercial (aPR, 95% CI: 0.44, 0.31–0.62) partners. Other factors associated with likely notification included perception of the partner as an STD source (aPR, 95% CI: 1.27, 1.10–1.48) and anticipated future sexual contact with the partner (aPR, 95% CI: 1.30, 1.11–1.52). An HIV diagnosis was associated with a lower likelihood of notification than non-HIV STDs (aPR: 0.68, 0.55–0.86). Qualitative discussion of the barriers and incentives to PN reflected a similar differentiation of anticipated notification according to partnership type and type of HIV/STD diagnosis.

**Discussion:**

Detailed attention to how partnership characteristics guide notification outcomes is essential to the development of new PN strategies. By accurately and thoroughly assessing the diversity of partnership interactions among individuals with HIV/STD, new notification techniques can be tailored to partner-specific circumstances.

## Introduction

Notification of sexual partners following a sexually transmitted disease (STD) diagnosis is a central component of public health efforts to control the spread of HIV and other STDs [1–4]. By focusing diagnosis and treatment efforts on the recent sexual contacts of persons newly diagnosed with HIV or another STD, partner notification (PN) offers the opportunity to target limited resources to the specific sexual networks that structure disease transmission in the larger population [5–7]. However, programs to support PN have had a limited effect on notification outcomes, with prior analyses estimating only 40–60% of named partners are notified worldwide [8–10]. Factors influencing notification outcomes include individual behavioral qualities (self-empowerment, depression, substance use), interpersonal partnership characteristics (partner type, partnership length, communication patterns), cultural contexts (HIV/STD stigma, community behavioral norms, structures of gender and sexuality), and socio-structural frameworks (access to testing and treatment services, local telecommunications infrastructure) [11–14]. In order to improve notification outcomes, improved understanding of the specific behavioral, social, and partnership contexts that guide PN in diverse international settings are needed.

Previous studies of PN have addressed the multilevel pathways that define notification outcomes. On the individual level, issues of self-efficacy and empowerment have been shown to influence notification behaviors [15–20]. On the partnership level, factors influencing PN include partnership type (primary/stable versus secondary/casual partners), as well as interpersonal dynamics (communication styles and relations of power within the partnership) [19–24]. In terms of biological factors, the type of STI diagnosed and the perceived likelihood of having infected (or having been infected by) a given partner also influence PN decisions [25–28]. Finally, social and structural characteristics including social norms of sexual behavior and communication, as well as the availability of infrastructural resources necessary to support partner notification and treatment, define larger contexts of PN practices [29, 30].

In Latin America, previous research on PN has indicated a need for new PN strategies [31–35]. In a survey of notification outcomes among 287 STD-positive, behaviorally high-risk men and women from urban Peru, 65% of respondents had notified their main partner within one year of their diagnosis, but only 10% had notified at least one of their casual partners [36]. While shame, embarrassment, and fear of rejection were reported as factors influencing decisions not to notify main and casual partners, lack of contact information was also commonly cited as a reason for non-notification of secondary partners. Qualitative research with men who have sex with men (MSM) and transgender women (TW) in Lima has provided further depth to these findings, suggesting that notification within primary partnerships is driven by a sense of responsibility and a desire to protect the health of both the individual and their main partner while notification in casual partnerships is frequently limited by the absence of detailed contact information, a lack of commitment within the partnership, and fear that stigmatizing information will be publicly disclosed [22].

However, these studies have either evaluated knowledge, attitudes, and practices among MSM/TW generally, or retrospectively reviewed notification outcomes among individuals previously diagnosed with HIV/STD. Few studies have addressed how the decision-making process at the point of diagnosis may influence subsequent notification practices. In order to design effective strategies to support PN, additional information is needed to assess and influence individual decision-making processes surrounding notification *as they occur*, i.e., at the point of HIV/STD diagnosis. To address this gap, we completed a mixed methods study of attitudes, beliefs, and anticipated partner notification practices among MSM and TW recently diagnosed with HIV and/or STD in Lima, Peru.

## Methods

Between January, 2011 and January, 2012 we enrolled 397 MSM and TW recently diagnosed with HIV, syphilis, genital herpes, genital ulcer disease (GUD), proctitis and/or urethritis. MSM and TW undergoing routine HIV/STD testing at the Asociación Civil Impacta Salud y Educación clinical research unit or the Alberto Barton municipal STD clinic, both in the Lima-Callao metropolitan area, were referred for screening by clinic staff. Enrollment was limited to men or TW who reported a recent male or transgender female sexual partner and who reported a new diagnosis of HIV, syphilis, genital herpes, genital ulcer disease (GUD), proctitis, and/or urethritis within the prior 30 days. After completing post-test counseling (which included standard partner notification recommendations), participants were invited to complete a survey about PN and compensated 10 *Nuevos soles* ($4 USD) for their transportation costs. Although participants were encouraged to complete the survey immediately following post-test counseling, they were allowed to return to complete the survey within a 30-day period, in order to accommodate potential emotional distress following HIV/STD diagnosis. All participants provided written informed consent prior to initiating any study procedures.

Participants completed an 82-item survey addressing demographics, HIV/STD history, attitudes related to PN, and characteristics of their three most recent sexual partners. Four-point Likert scales were used to address general attitudes about the importance of PN, norms of notification among their peers and partners, likelihood of notifying their three most recent partners, and reasons why each partner would or would not be notified. For the purpose of data analysis, 4-point Likert scale responses (Very Likely/Somewhat Likely/Somewhat Unlikely/ Very Unlikely) were re-categorized into binary (Likely/Unlikely) outcomes.

A subset of 30 participants was selected to participate in brief qualitative interviews to explore in greater depth issues addressed in the quantitative survey. Interview participants were recruited from the Barton STD clinic site only and were sampled by convenience, according to participant and interviewer availability. (There were no statistically significant differences between the subset of participants interviewed and the larger study population.) No additional compensation was provided for participating in the qualitative interview. Interviews lasted 15–20 minutes and used a semi-structured script designed to elaborate on answers provided in the quantitative survey. Interviews were audio-recorded, transcribed verbatim, and coded by two separate readers. Following initial review, a preliminary codebook of major themes was developed and used to systematically code transcripts. The codebook was reviewed and revised in an iterative process, with discrepancies between coders resolved through discussion and consensus, in order to ensure reliability and accuracy throughout the data analysis process. Themes were compiled and developed into concepts through collaborative discussion between members of the study team. All participant quotations are identified by pseudonyms created for the purpose of this study.

### Ethics, Consent, and Permissions

The study was approved by the Institutional Review Boards of the University of California, Los Angeles (G10-03-036-01) and Asociación Civil Impacta Salud y Educación (0104-2010-CE). Written informed consent was obtained from all participants prior to enrollment.

### Data Analysis

All analyses were limited to participants who provided information on at least one notifiable (non-anonymous) male sexual partner. Demographic and behavioral characteristics of the population, and general attitudes, beliefs and practices related to PN were characterized by prevalence estimates for categorical data, or medians for continuous data. Likely notification of each respondent’s three most recent partners was used to estimate the frequency of anticipated notification and assess participant- and partner-specific factors associated with anticipated PN. Due to the small number of female partners reported, only male and transgender female partners were included in the analysis. Anonymous partners (“Someone you have had sex with but don’t know their name or other identifying information”) were excluded from all partner-specific analyses since anonymous partners, by definition, could not be contacted or notified.

To assess partner-specific attitudes and anticipated notification behavior, we used prevalence ratios (PR) to analyze participant- and partner-level characteristics associated with anticipated likelihood of notifying each of the participants’ three most recent partners. For each partner reported, participants were asked whether they were likely to notify this specific partner. Participant-level characteristics evaluated include age, education level, sexual identity, sexual role, number of sexual partners in the previous three months (with continuous data re-categorized as 1, 2–3, or ≥4 partners), and type of STD diagnosis (HIV only, HIV and another STD, or other STD only). Partner-level factors assessed in the analysis were partnership type (stable, casual, or commercial), partner sexual identity (as described by the participant), history of unprotected anal intercourse (UAI) with the partner, perceived likelihood of having been infected with an STD by the partner, and anticipated likelihood of future sexual contact with the partner.

Crude and adjusted prevalence ratios, along with their 95% confidence intervals, were computed for participant- and partner-level factors associated with anticipated notification using Poisson regression with robust variance estimation and GEE extension with an exchangeable correlation structure [37, 38]. GEE was used to account for the correlation of variables (both outcome and several predictors) measured at the partnership level (lower-level unit), where a maximum of three recent partnerships were reported by each study participant (upper-level unit) [38]. Only partnerships with complete data (622 of 844 partnerships reported) were included in the final adjusted regression model. All statistical analyses were completed using Stata 11.0 (Stata Corporation, College Station, TX).

## Results

We enrolled 397 MSM and TW recently diagnosed with HIV, syphilis, genital herpes, genital ulcer disease (GUD), proctitis, and/or urethritis, of whom 350 provided information on at least one recent, notifiable male partner. Demographic information and STD prevalence data is provided in Table 1. Most subjects included in the analysis defined their sexual identity as gay or homosexual (66.3%) and their sexual role during intercourse as *pasivo* (receptive; 38.9%) or *moderno* (versatile; 46.7%). The majority of participants had been recently diagnosed with syphilis (53.4%) and/or HIV infection (48.6%), with smaller numbers reporting a diagnosis of urethritis/proctitis (13.1%) or genital herpes (9.1%). Information on 844 sexual partnerships with male or transgender female partners was provided, most of which were with casual (48.9%) or stable (35.6%) partners and the remainder with commercial sex workers (0.8%) or clients (14.7%). Participants most often identified their partners as gay/homosexual (43.4%) or bisexual (40.1%) and their sexual role during intercourse as *activo* (insertive; 53.1%) or *moderno* (versatile; 31.6%).

**Table 1:** Participant and Partner Characteristics of MSM/TW Recently Diagnosed with HIV and/or STI; Lima, Peru 2011.

**Participant Characteristics**		**(N = 350 Participants)**
Age	Median (IQR)	28 (24–35)
Education	High School Graduate	127 (36.4%)
	University/Vocational Training	143 (41.0%)
Sexual Identity (n = 344)	Heterosexual	11 (3.2%)
	Bisexual	54 (15.7%)
	Homosexual	228 (66.3%)
	Transgender	51 (14.8%)
Sexual Role (n = 347)	*Activo* (Insertive)	50 (14.4%)
	*Pasivo* (Receptive)	135 (38.9%)
	*Moderno* (Versatile)	162 (46.7%)
HIV/STI Diagnosis (n = 350)	HIV	81 (23.2%)
	HIV/STI Co-Infection	89 (25.4%)
	Non-HIV STI	180 (51.4%)
Specific STI(s) Diagnosed (n = 350)	HIV	170 (48.6%)
	Syphilis	187 (53.4%)
	Gonorrhea/Chlamydia	46 (13.1%)
	HSV-2	32 (9.1%)
Number of Male Sex Partners (3 Months) (n = 350)	1	71 (20.3%)
	2 or 3	84 (24.0%)
	≥4	195 (55.7%)
**Partner Characteristics**		**N = 844 Partners**
Type of Partner (n = 844)	Stable	300 (35.6%)
	Casual	413 (48.9%)
	Commercial Sex client	124 (14.7%)
	Commercial Sex Worker	7 (0.8%)
Partner Sexual Identity* (n = 786)	Heterosexual	112 (14.2%)
	Bisexual	315 (40.1%)
	Homosexual	341 (43.4%)
	Transgender	18 (2.3%)
Partner Sexual Role*	*Activo* (Insertive)	440 (53.1%)
(n = 829)	*Pasivo* (Receptive)	127 (15.3%)
	*Moderno* (Versatile)	262 (31.6%)
UAI with Partner (Insertive and/or Receptive) (n = 705)		304 (42.5%)
Partner Likely Source of STI (n = 715)		227 (27.0%)
Anticipate Future Sexual Contact with Partner (n = 388)		443 (52.9%)

*As described by the participant

Participants anticipated notifying approximately half (52.5%; 443/844) of all non-anonymous partners (Table 2). Partnership type was associated with anticipated notification, with 78.3% (235/300) of stable partners likely to be notified, compared with 43.8% (181/413) of casual and 20.6% (27/131) of commercial partners (p<0.05 for both comparisons). Notification was also more likely for partners with whom participants anticipated future sexual contact (adjusted Prevalence Ratio and 95% Confidence Interval [aPR, 95% CI] = 1.30, 1.11–1.52) or who were perceived as likely to have been the source of infection (aPR, 95% CI = 1.27, 1.10–1.48) (Table 3). Participant characteristics associated with likely notification included the number of recent male sex partners reported and the type of STD diagnosed, with anticipated notification less likely among participants with 4 or more recent partners (aPR, 95% CI = 0.81, 0.67–0.97) and among participants diagnosed with HIV compared with another STD (aPR, 95% CI = 0.68, 0.55–0.86).

**Table 2:** Incentives and Barriers to Partner Notification (By Partner Type) Among MSM/TW Recently Diagnosed with HIV and/or STD; Lima, Peru 2011.

	Partner Type		
	Main (N = 300)	Casual (N = 413)	Commercial (N = 131)
**Reasons for Notification**			
**Total Partners Anticipated to Be Notified**	**n = 235 (78.3%)**	**n = 181 (43.8%)**	**n = 27 (20.6%)**
To Protect Participant’s Health	189 (80.4%)	116 (64.1%)	10 (37.0%)
To Protect Partner’s Health	125 (53.2%)	100 (55.2%)	22 (81.5%)
To Protect Community Health	72 (30.6%)	56 (30.9%)	9 (33.3%)
To Maintain Trust in Partnership	125 (53.2%)	76 (42.0%)	10 (37.0%)
**Reasons Against Notification**			
**Total Partners Anticipated To Not Be Notified**	**n = 65 (21.7%)**	**n = 232 (55.9%)**	**n = 104 (79.4%)**
Fear of Rejection by Partner	65 (100.0%)	86 (37.1%)	20 (19.2%)
Fear of Violence from Partner	39 (60.0%)	79 (34.0%)	29 (27.9%)
Fear of Gossip in Community	26 (40.0%)	72 (31.0%)	13 (22.1%)
No Contact Information for Partner	21 (32.3%)	54 (23.3%)	44 (42.3%)
Not Important to Notify Partner	18 (27.7%)	62 (26.7%)	19 (18.3%)

**Table 3:** Participant and Partner Characteristics Associated with Anticipated Partner Notification Among MSM/TW Recently Diagnosed with HIV and/or STD; Lima, Peru 2011.

**Participant Characteristics**						
		**N**	**cPR (95%CI)**	**p**	**aPR (95%CI)**	**p**
Age	Years	348	1.00 (0.99–1.01)	0.30	1.00 (0.99–1.01)	0.72
Education	Non-High School Graduate	79	Ref	Ref	Ref	Ref
	High School Graduate	127	1.13 (0.88–1.46)	0.33	1.01 (0.79–1.28)	0.94
	University/Vocational Training	143	1.32 (1.04–1.67)	<0.05	1.13 (0.91–1.41)	0.27
Participant Sexual Role	*Activo*	50	Ref	Ref	Ref	Ref
	*Pasivo*	135	0.63 (0.50–0.79)	<0.05	0.82 (0.65–1.03)	0.09
	*Moderno*	162	0.82 (0.68–1.00)	0.06	0.94 (0.77–1.15)	0.56
Type of HIV/STD Diagnosis	Non-HIV STD	180	Ref	Ref	Ref	Ref
	HIV	81	0.67 (0.53–0.86)	<0.05	**0.68 (0.55–0.86)**	**<0.05**
	HIV/STD Co-Infection	89	0.93 (0.77–1.12)	0.48	0.86 (0.72–1.04)	0.13
Number of Male Sexual Partners (3 Months)	1	71	Ref	Ref	Ref	Ref
	2–3	84	0.82 (0.68–1.00)	0.06	0.86 (0.71–1.04)	0.12
	≥4	195	0.63 (0.53–0.75)	<0.05	**0.81 (0.67–0.97)**	**<0.05**
**Partner Characteristics**							
		n/N*	**cPR (95%CI)**	**p**	**aPR (95%CI)**	**p**
Partner Type	Stable	235/300	Ref	Ref	Ref	Ref
	Casual	181/413	0.58 (0.50–0.68)	<0.05	**0.63 (0.54–0.75)**	**<0.05**
	Commercial	27/131	0.33 (0.24–0.46)	<0.05	**0.44 (0.31–0.62)**	**<0.05**
Partner Sexual Identity	Hetero/Bisexual	204/427	Ref	Ref	Ref	Ref
	Homosexual	206/341	1.23 (1.04–1.45)	<0.05	1.08 (0.90–1.29)	0.41
	Transgender	13/18	1.57 (1.08–2.29)	<0.05	1.12 (0.77–1.82)	0.43
UAI	No UAI with Partner	176/411	Ref	Ref	Ref	Ref
	UAI with Partner	189/304	1.47 (1.26–1.71)	<0.05	1.11 (0.96–1.29)	0.15
Perceived STI Source	Partner Not Perceived as Likely Source of HIV/STD	290/613	Ref	Ref	Ref	Ref
	Partner Perceived as Likely Source of HIV/STD	152/227	1.35 (1.17–1.55)	<0.05	**1.27 (1.10–1.48)**	**<0.05**
Future Sexual Contact	Future Sexual Contact with Partner Not Anticipated	160/395	Ref	Ref	Ref	Ref
	Future Sexual Contact with Partner Anticipated	278/343	1.58 (1.35–1.86)	<0.05	**1.30 (1.11–1.52)**	**<0.05**

*Due to clustering of data at participant-level, n/N reported for Partner Characteristics only

Qualitative analysis mirrored the general attitudes as well as incentives/barriers to notification observed in the quantitative data (Table 4). Partnership type significantly shaped participants’ attitudes, with primary responsibility for notification seen towards main partners and less importance placed on notification of casual partners. Open communication and trust were frequently cited as important factors in deciding whether to notify a primary partner, “because they love each other, they live together and share many things… I think that in more stable partnerships you talk more than in a casual encounter.” (Rafael; Gay, HIV) In this context, PN was considered something both important and inevitable, “because sooner or later they are going to find out, and better if, before she finds out from other sources, that I tell her myself.” (Ivan; Bisexual, Urethritis) In contrast, anticipated notification of casual partners was infrequent and justified by the individual’s perceived likelihood of having transmitted an STI during their sexual contact such that, “I don’t think I will inform him because it was all with protection, except for the oral sex. There is a risk there, but I’m being sincere that right now I don’t have—I don’t think I will tell him.” (Aldo; Heterosexual, Urethritis) The lack of interpersonal trust and honesty observed in casual partnerships often translated into a type of fatalism or lack of concern for the potential consequences of HIV or STI transmission where, “In the case of the person who infected me, I don’t think it’s important to tell him because when I asked him, ‘Have you been tested?’ he told me, ‘Yes, one month ago,’ ‘What did they tell you?’ ‘That I didn’t have anything,” and I don’t believe him.” (Fernando; Gay, HIV and Syphilis) Similarly, for an anonymous partner, “I don’t place a lot of importance on it because it was an anonymous partner, so he knew that what could happen, could happen.” (Oscar; Gay, Syphilis) Limited availability of contact information also precluded notification of many casual and commercial partners since, “I don’t know where to find them. It was, as you say, a casual relation. I found them in the street, on some avenue.” (Enrique; Gay, Syphilis)

**Table 4:** Qualitative Analysis of Perceptions, Incentives, and Barriers to Partner Notification Among MSM/TW Recently Diagnosed with HIV/STI; Lima, Peru 2011.

**Partner Notification: General Perceptions**	
**Stable Partners**	“There is a greater responsibility when you live with someone or you have a longer time together in your sexual relations… with a stable partner there’s more responsibility to communicate with them about what’s going on.” (Enrique; Gay, Syphilis)
**Casual Partners**	“I think that it’s important to tell your partner. Because in that way, he is informed and can take the necessary steps…. I’m referring to a main partner, because a casual, you see them one time and then you never see them again.” (Julian; Gay, Proctitis)
**Anonymous Partners**	“I would never tell them, simply because they are “hit and runs” [*choque y fugas*], and I will never see them again.” (Elian; Heterosexual, Urethritis/GUD)
	“I was with him in the movie theater [*cine*]. So with him, I don’t think I will see him again, so I don’t think I will tell him.” (Alejandro; Heterosexual, Proctitis)
**Commercial Partners**	“If a guy finds out that you infected him, one of your clients, then you are already done for [*ya fuiste*] and he is going to come looking for you, because you burned him [*lo has quemado*].” (Cristina; Transgender, HIV)
**Female Partners**	“Because it’s more difficult to tell a partner, in this case bisexual, that I was with a man, than to tell a man I was with a woman.” (Jose; Bisexual, HIV/Syphilis)
	“For the fear that they are going to say to you, ‘What are you doing with men?’” (Carlos; Heterosexual, HIV)
**Incentives to Notification**	
**Protect Self**	“So that he can get tested and see if he’s okay. That way we can both know that we’re well, we’re healthy. Because, apparently, someone can look healthy and you don’t know how they are really.” (Javier; Gay, Urethritis)
**Protect Partner**	“It’s a question of mutual care and, if I love him, I have to care for him like I believe he would do with me.” (Jose; Bisexual, HIV/Syphilis)
**Maintain Trust**	“It’s more important to talk with your partner, so that they trust you. We all make mistakes, and if you tell them in time they may understand.” (Ivan; Bisexual, Urethritis)
	“With a main partner, you share things, you achieve a level of trust—in quotations, ‘trust’—a level of ‘trust’ so maybe you can tell them.” (Aldo; Heterosexual, Urethritis)
**Barriers to Notification**	
**Fear of Rejection**	“For me, it’s important to tell my partner, my true partner, but it makes me a little afraid of rejection. Because of that ‘Where did it come from?’ It came from being with other partners, and she is going to think the worst, she is going to throw me out.” (David; Heterosexual, HIV)
	“It’s a fear, fear of losing that contact, their friendship, or whatever. It’s a fear.” (Aldo; Heterosexual, Urethritis)
**Fear of Violence**	“I don’t know him well, and I am a little afraid to tell him. Because I see him around, he works in the street, he has a group that’s, let’s say, very “rough” [*movido*]. That scares me, that maybe he will react with violence.” (Miguel; Gay, HIV)
	“My fear is always that, ‘How can I say I have HIV? How are you going to react? Will you grab me and punch me, or hit me, or will you cut me off? What will you do, what will be your reaction?” (Cristina; Transgender, HIV)
**Fear of Stigma**	“That he would reject me… or that he would say when he sees me, ‘That faggot [*maricon*] is like that,’ that he would say to me that I’m a disease carrier, or that he would say it to someone else.” (Scarlet; Transgender, HIV)
	“Because there is no one trustworthy and I can’t confide in anyone other than my family. And I know that sometimes there are these little arguments, resentments, I tell someone and we fight, and so he tells another person, and another, and another, so then the whole world finds out.” (Princesa; Transgender, HIV)
**Lack of Contact Information**	“I’m not going to tell him because it’s unlikely that I will see him again. Or maybe I will see him, but most likely, I won’t.” (Oscar; Gay, Syphilis)
	“I don’t know where to find them. It was, as they say, a casual relationship, I met them on the street, on some avenue.” (Enrique; Gay, Syphilis)
**Loss of Income**	“Because, really, you need to work. At the moment you tell them, they are going to reject you and won’t ever want to come near you.” (Scarlet; Transgender, HIV)

Motivations and hindrances to notification followed a similar pattern, with PN described as both a way to strengthen trust within a relationship and as a potential threat to the stability of the partnership. Many participants described notification as a way to maintain trust and protect the health of both partners in a committed relationship: “I will tell him, and I will tell him so we can care for each other and because he loves me, and so that gives me the trust to tell him.” (Cristina; Transgender, HIV) At the same time, fear of rejection, disclosure of sexual infidelity, and disclosure of same-sex sexual activity were cited as key obstacles to notification within primary partnerships such that, “It’s harder to tell your lady. With the other person, you can say, ‘Hey, look, you infected me with something,’ because the other person could have been with someone else. But your stable partner, no.” (Oswaldo; Heterosexual, Urethritis) Although the possibility of intimate partner violence was often cited as a concern, fear that disclosure would end a relationship was also frequently reported, “not the fear that he could touch me, or hit me, no. Fear about how he could react, and maybe you break ties.” (Aldo; Heterosexual, Urethritis) The bidirectional nature of these fears was reflected in one participant’s assessment of whether his recent partners would tell him if they were diagnosed with an STI: “They would never tell me. They would never tell me because of fear of cutting off the friendship.” (Carlos; Heterosexual, HIV)

On a community-level, participants often articulated concerns that disclosure of an HIV/STD diagnosis would lead to their being publicly identified as promiscuous or diseased, reinforcing negative cultural stereotypes about MSM and TW in Peru: “Here in Lima, generally, it is believed that there is a lot of promiscuity among homosexuals, even more so among *pasivo* [receptive] homosexuals. I feel like there is a lot of prejudice, and so the fact of notifying someone contributes to those prejudices.” (Javier; Gay, Urethritis) Unfortunately, this societal stigma against STDs not only inhibited notification but also precluded the public dissemination of accurate knowledge concerning HIV and STD prevention, diagnosis and treatment: “People speak in murmurs, nothing more, ‘This kid has this and that,’ but there is nowhere that they offer you a solution, that they tell you like a doctor does, or like you are telling me now, what is herpes, what is syphilis, gonorrhea… that there is treatment.” (Cristina; Transgender, HIV)

## Discussion

Our analysis provides important information on the attitudes, beliefs, and anticipated practices related to partner notification among MSM/TW in Lima, Peru newly diagnosed with HIV and/or another STD. By interviewing participants during the time period immediately after receiving their diagnosis, we were able to assess the specific factors influencing their PN decision-making processes at the time these decisions were being made. As in previous research with similar populations, partnership type significantly shaped PN beliefs and practices, with main partners more likely to be notified than casual or commercial partners, and for distinct reasons [24, 39]. As a result, detailed understanding of how anticipated PN practices vary between different partnership contexts provides an important area for future research to improve notification outcomes.

The significant difference in anticipated notification of primary/stable partners compared with secondary/casual and commercial sex partners observed in our sample reflects the primacy of partnership type in structuring PN decision-making processes and practices. As in previous studies in Peru and other global contexts, partnership type not only influences likelihood of PN but also shapes perceptions of potential risks and benefits of notification [26, 40]. For main partners, the most commonly cited reasons for PN were to protect the health of both the participant and their partner, and to maintain trust in the relationship. Similarly, the most frequently endorsed explanations for non-notification were intimate partnership issues including fear of rejection and violence [41, 42]. Qualitative findings supported these observations, emphasizing the importance of trust, communication, and mutual care within primary partnerships, and the risk for potential disclosure of sexual infidelity in revealing an STD diagnosis [43]. In contrast, for casual and commercial partners, notification decisions were based less on participants’ concern for their own health and more oriented towards protecting the health of the partner (possibly because they did not anticipate future sexual contact with these individuals and so predicted a lower personal risk for re-exposure than with a recurrent partner). For these contacts, perceived barriers to notification were evenly distributed between different justification options, with perceived likelihood of transmission emphasized and fears of rejection and violence far less prominent than with main partners.

The other associations with anticipated PN we observed (e.g., the participant’s number of recent sexual partners, the likelihood of future sexual contact with a given partner, and the perception of a given partner as the likely source of infection) all align with the differences noted according to partnership type. Participants with higher numbers of recent partners (≥4 in 3 months) were less likely to maintain a single, stable partnership in favor of multiple casual and/or commercial partners, and subsequently were also less likely to anticipate notifying these recent partners. Similarly, participants who did not anticipate ongoing sexual contact with a regular partner were less likely to anticipate notifying the person of their diagnosis. Highlighting the importance of protecting the participant against STD re-infection, regardless of their partnership status, and potentially ascribing blame for STD transmission, participants who believed that a recent partner was likely to have been the source of their infection were significantly more likely to anticipate notifying that person [21, 44].

Aside from partnership characteristics, the other key factor influencing notification decisions was whether the participant had been diagnosed with HIV as compared with another STD [45]. The lower frequency of anticipated notification for HIV compared with a non-HIV STD diagnosis reflects the high degree of stigma and shame associated with HIV infection in Peru. In previous qualitative research, MSM in Peru considered HIV infection as heavily stigmatized, and described the possibility of notifying a partner of an HIV diagnosis outside their frame of reference [43]. This observation was reinforced by HIV-infected participants who described fears of interpersonal violence, social exclusion, and societal stigma as important factors hindering potential notification.

Our data highlight the interaction of individual attitudes and perceptions, interpersonal partnership factors, biological STD characteristics, and social and cultural structures in shaping partner-specific notification practices. While key criteria like partnership type serve as organizing paradigms for participants’ attitudes and anticipated notification practices, an array of other factors also contribute to partner-specific notification decisions. Similarly, the motivations and barriers influencing notification outcomes were primarily guided by partnership type and reflected a complicated calculus of individual, interpersonal, and social risks and benefits. Most importantly, this diversity of partnership types, incentives/barriers to notification, and anticipated notification practices was simultaneously observed within individual participants at the time of diagnosis. As a result, effective PN systems will need to acknowledge and address the multiplicity of PN contexts, decision-making processes, and potential outcomes frequently encountered by individual patients at the time of HIV/STD diagnosis [46].

Our data has several characteristics that limit the generalizability of our findings. Since the analysis focuses on anticipated partner notification, we do not have information on actual notification outcomes, though the frequency of anticipated notification was similar to the prevalence of actual notification reported in a similar population from urban Peru [36]. As a result, participant responses are subject to influence from a range of factors, including social desirability bias, and may not accurately reflect their actual likelihood of PN. By enrolling a convenience sample of individuals recently diagnosed with HIV and/or STD, and without collecting information on the refusal rate of potential participants invited to complete the survey, we cannot determine whether participants in our study are representative of the larger MSM/TW population. Similarly, our study was not designed to explore differences between how subgroups of MSM and TW (such as between cis-gender men and transgender women, or between men with exclusively male partners and men with male and female partners) may understand and approach central issues of PN differently. However, by analyzing both quantitative and qualitative data from MSM/TW at the time of their HIV/STD diagnosis, our findings provide a unique source of important information on the individual, interpersonal, and social factors that structure anticipated PN decisions among MSM and TW in Lima, Peru.

We present data on anticipated PN practices among Peruvian MSM and TW newly diagnosed with HIV and/or another STD. Our findings highlight the importance of partnership type and associated characteristics in structuring PN decisions and practices. Detailed attention to how partnership characteristics (including partner type, frequency of sexual contact, trust and communication within the relationship) define incentives and barriers to notification, and ultimately guide notification outcomes, is essential to the development of new PN strategies. Given the availability of new and traditional PN technologies, including patient-delivered referral cards, expedited partner therapy, anonymous internet-based notification systems, and third-party contact tracing, the diverse range of available resources can be adapted to the specific variations in partnership interactions that underlie patients’ anticipated notification practices [47–49]. In contrast to the “one size fits all” public health approaches to PN common in developing countries, new approaches to PN should pay detailed attention to how an individual’s range of recent partnership characteristics, their partner-specific patterns of communication, and social and structural factors collectively promote or discourage partner-specific notification practices [29]. By accurately and thoroughly assessing the diversity of partnership interactions maintained by individuals with an HIV/STD diagnosis, new notification technologies can then be tailored to the circumstances where they are likely to have the greatest effect.

## Supporting Information

S1 File Quantitative survey instrument. (DOCX)

S2 File Qualitative semi-structured interview script. (DOCX)
