# Supplementary material for: Anticipated Notification of Sexual Partners following STD Diagnosis among Men Who Have Sex with Men and Transgender Women in Lima, Peru: A Mixed Methods Analysis
Source: PLoS One. 2016 Sep 29;11(9):e0163905. doi: 10.1371/journal.pone.0163905 (PMC5042523; doi:10.1371/journal.pone.0163905)
Supplement: S1 File — (DOCX) [file pone.0163905.s001.docx]

**S1 File. Quantitative Survey. Quantitative survey instrument.**

**Partner Notification Survey-Version 1.1**

**Thank you for taking the time to complete this survey. Please read and answer every question carefully. If you do not know the answer to a question, please respond, “I don’t know.” If you are not comfortable or prefer not to answer a specific question, please respond, “No answer.”**

Participant Study ID:

1. How old are you (age in years)?
   1. __________
   2. I don’t know
   3. No answer
2. In what district do you live?
   1. __________
   2. I don’t know
   3. No answer
3. What is the highest level of education you have completed?
   1. No formal education
   2. Elementary/Primary School
   3. Some High School/Secondary School
   4. Graduated High School/Secondary School
   5. Some University or Professional School
   6. Graduated University or Professional School
   7. I don’t know
   8. No answer
4. What is your sexual identity?
   1. Heterosexual
   2. Bisexual
   3. Homosexual
   4. Transgender
   5. Other:_______________
   6. I don’t know
   7. No answer
5. What is your sex role during intercourse?
   1. Activo
   2. Pasivo
   3. Moderno
   4. Other:______________
   5. I don’t know
   6. No answer
6. In the past month, have you been told you have any of the following sexually transmitted infections (STIs):

(Note: For these questions, we are only asking about a diagnosis that was made by a doctor or other health professional based on the results of a blood test or other laboratory test. We are not asking about a diagnosis made by a pharmacist based on symptoms or complaints, and we are not asking about whether a friend or partner told you that you might have one of these infections.)

- 1. HIV (the virus that causes AIDS)
     1. Yes
     2. No
     3. I don’t know
     4. No answer
  2. Syphilis
     1. Yes
     2. No
     3. I don’t know
     4. No answer
  3. Gonorrhea or Chlamydia
     1. Yes
     2. No
     3. I don’t know
     4. No answer
  4. Genital or Anal Herpes
     1. Yes
     2. No
     3. I don’t know
     4. No answer
  5. Genital or Anal Warts
     1. Yes
     2. No
     3. I don’t know
     4. No answer
  6. Another sexually transmitted infection (STI)
     1. Yes (Please specify:_____________________)
     2. No
     3. I don’t know
     4. No answer

1. **In the past month,** how many different partners (men and women) have you had (how many different people have you had sex with)?
   1. __________
   2. I don’t know
   3. No answer

(If Zero, skip to Question 10)

1. In the past month, how many of your partners have been men (or male-to-female transgender)?
   1. __________
   2. I don’t know
   3. No answer
2. In the past month, how many of your partners have been women (not transgender)?
   1. __________
   2. I don’t know
   3. No answer
3. **In the past three months,** how many different people have you had sex with?
   1. __________
   2. I don’t know
   3. No answer

(If Zero, skip to Question 17)

1. In the past three months, how many of your partners have been men (or male-to-female transgender)?
   1. __________
   2. I don’t know
   3. No answer

(If zero, skip to Question 14)

1. In the past three months, how many men did you have unprotected insertive anal sex with (you penetrated them without a condom)?
   1. __________
   2. I don’t know
   3. No answer
2. In the past three months, how many men did you have unprotected receptive anal sex with (they penetrated you without a condom)?
   1. __________
   2. I don’t know
   3. No answer
3. In the past three months, how many of your partners were women?
   1. ________________
   2. I don’t know
   3. No answer

(If Zero, skip to Question 17)

1. In the past three months, how many women did you have unprotected vaginal sex with (you penetrated them without a condom)?
   1. __________
   2. I don’t know
   3. No answer
2. In the past three months, how many women did you have unprotected anal sex with (you anally penetrated them without a condom)?
   1. __________
   2. I don’t know
   3. No answer
3. **In the past three months,** have you had a “primary” partner (i.e., someone you are in a long-term or stable relationship with such as a husband, wife, partner, boyfriend, or girlfriend)?
   1. Yes
      1. In the past three months, how many primary partners have you had?

_______________

- 1. No
  2. I don’t know
  3. No answer

1. In the past three months, have you had a “secondary” partner (i.e., someone who you know and have had sex with once or on multiple occasions, but who you do not maintain a stable relationship)
   1. Yes
      1. In the past three months, how many secondary partners have you had?

_________________

- 1. No
  2. I don’t know
  3. No answer

1. In the past three months, have you had an “anonymous” partner (i.e., someone who you had sex with but didn’t know their full name)?
   1. Yes
      1. In the past three months, how many anonymous partners have you had?

_________________

- 1. No
  2. I don’t know
  3. No answer

1. In the past three months, have you had a “commercial” partner (i.e., someone that you have sex with in exchange for money, food, clothing, shoes, or other goods)?
   1. Yes
      1. In the past three months, how many partners have you had that **you** **gave** money or other goods in exchange for sex?_________________
      2. In the past three months, how many partners have you had that **gave** **you** money or other goods in exchange for sex?_________________
   2. No
   3. I don’t know
   4. No answer

*The next set of questions is going to address the topic of “Partner Notification.” When we say Partner Notification, we are talking about telling the people you have had sex with that you have HIV, syphilis, or another STI. In the following questions we will be asking about “main, or primary,” partners and “casual, or secondary,” partners. By “main” partner we mean someone with whom you have a stable, ongoing relationship like a boyfriend/girlfriend, husband/wife, or spouse/partner. By “casual” partner we mean someone who you have had sex with at least once but whom you do not have a stable or ongoing relationship, such as a “punto” or a friend.*

1. How important do you think it is for people to notify their main or primary partner(s) when they have been diagnosed with an STI (like HIV or syphilis)?
   1. Extremely important
   2. Very important
   3. Somewhat important
   4. Not important
   5. I don’t know
   6. No answer
2. Do you think that it is important for people to notify their casual or secondary partner(s) when they have been diagnosed with an STI (like HIV or syphilis)?
   1. Yes, very important
   2. Yes, somewhat important
   3. No, not very important
   4. No, not at all important
   5. I don’t know
   6. No answer
3. How many of your friends would tell their main partner(s) that they were diagnosed with an STI (like HIV or syphilis)?
   1. All of my friends would tell their main partner
   2. Most of my friends would tell their main partner
   3. About half of my friends would tell their main partner
   4. Some of my friends would tell their main partner
   5. None of my friends would tell their main partner
   6. I don’t know
   7. No answer
4. How many of your friends would tell at least one of their casual partner(s) that they were diagnosed with an STI (like HIV or syphilis)?
   1. All of my friends would tell their casual partners
   2. Most of my friends would tell their casual partners
   3. About half of my friends would tell their casual partners
   4. Some of my friends would tell their casual partners
   5. None of my friends would tell their casual partners
   6. I don’t know
   7. No answer
5. How many of your recent partner(s) (the people you have had sex with in the past three months) do you think would tell you if they were diagnosed with an STI like HIV or syphilis?
   1. All of my partner(s) would tell me about their diagnosis
   2. Most of my partner(s) would tell me about their diagnosis
   3. About half of my partner(s) would tell me about their diagnosis
   4. I don’t believe that any of my partner(s) would tell me if they were diagnosed with an STI
   5. I don’t know
   6. No answer
6. If there existed an internet website that you could use to send anonymous email messages to your partners to tell them that they might have been exposed to HIV, syphilis, or another STI, how likely would you be to use it?
   1. I would definitely use it
   2. I would probably use it
   3. I would probably not use it
   4. I would definitely not use it
   5. I don’t know
   6. No answer
7. If there existed an internet website that you could use to send anonymous email messages to your partners to tell them that they might have been exposed to HIV, syphilis, or another STI, how likely would you be to use it with each of the following types of partners?
   1. Main or primary partners
      1. Extremely likely
      2. Somewhat likely
      3. Somewhat unlikely
      4. Extremely unlikely
      5. I don’t know
      6. No answer
   2. Casual or secondary partners
      1. Extremely likely
      2. Somewhat likely
      3. Somewhat unlikely
      4. Extremely unlikely
      5. I don’t know
      6. No answer
   3. Commercial sex partners
      1. Extremely likely
      2. Somewhat likely
      3. Somewhat unlikely
      4. Extremely unlikely
      5. I don’t have any commercial sex partners
      6. I don’t know
      7. No answer

**We are now going to ask you a series of questions about your three most recent sex partners. It is important that you answer questions about the three people you have recently had sex with most recently, in chronological order, and not just the partners with whom you have a relationship. Remember that all of your answers are confidential and anonymous.**

**Thinking about the last person you had sex with (Partner #1),**

1. Was this person (choose the best option):
   1. A stable, main, or primary partner
   2. A casual or secondary partner
   3. An anonymous partner
   4. A sex work client
   5. A sex worker
   6. I don’t know
   7. No answer
2. Was this person:
   1. Male
   2. Female
   3. Transgender
   4. I don’t know
   5. No Answer
3. (For male partners) What do you think is this partner’s sexual identity?
   1. Heterosexual
   2. Bisexual
   3. Homosexual
   4. Transgender
   5. Other:_______________
   6. I don’t know
   7. No answer
4. (For male partners) What do you think is this partner’s sexual role?
   1. Activo
   2. Pasivo
   3. Moderno
   4. Other:______________
   5. I don’t know
   6. No answer
5. How many times have you had sex with this partner?
   1. 1 time
   2. 1-3 times
   3. 3-10 times
   4. More than 10 times
   5. I don’t know
   6. No answer
6. When was the last time you had sex with this partner?
   1. Less than one week ago
   2. Less than one month ago
   3. Less than three months ago
   4. Less than six months ago
   5. More than six months ago
   6. I don’t know
   7. No answer
7. Do you think you will have sex with this partner again in the future?
   1. I will definitely have sex with this partner again in the future
   2. I will probably have sex with this partner again in the future
   3. I will probably not have sex with this partner again in the future
   4. I will definitely not have sex with this partner again in the future
8. **During the past three months,** what sexual practices did you engage in with this partner?
   1. You performed oral sex on them?

Y/N With a condom? Y/N

- 1. They performed oral sex on you?

Y/N With a condom? Y/N

- 1. They penetrated you?

Y/N With a condom? Y/N

- 1. You penetrated them (anal or vaginal intercourse)?

Y/N With a condom? Y/N

- 1. Other sexual acts? Y/N
     1. If so, please specify:___________________________________
  2. I don’t know
  3. No answer

1. How likely is it that you transmitted your STI to this partner?
   1. Not at all likely or Impossible
   2. Somewhat unlikely
   3. Somewhat likely
   4. Very likely or certain
   5. I don’t know
   6. No answer
2. How likely is it that this partner infected you (i.e., that they are the source of your STI)?
   1. Not at all likely or Impossible
   2. Somewhat unlikely
   3. Somewhat likely
   4. Very likely or certain
   5. I don’t know
   6. No answer
3. Do you know how to contact this partner?
   1. In person (i.e., you know where they live or where they socialize)? Y/N
   2. By telephone Y/N
   3. By e-mail Y/N
   4. By Instant Messenger (e.g., MSN Messenger, Yahoo Messenger, IM, etc.) Y/N
   5. By another means? Y/N
      1. If so, please specify_________________________________________
   6. I don’t know
   7. No answer
4. Do you plan to tell (or have you already told) this partner about your diagnosis?
   1. Yes, I have already told this partner
   2. Yes, I will definitely tell this partner
   3. Yes, I will probably tell this partner

If A, B, or C:

- - 1. What are some of the reasons why you plan to notify (or notified) this partner (check all that apply):
       1. To protect my partner’s health
       2. To protect my health
       3. To protect the health of men in my community
       4. To maintain trust in our relationship
       5. Other:______________________
    2. What is the most likely way you would notify (or notified) this partner (check the best option)?
       1. In person
       2. By telephone
       3. By e-mail
       4. By Instant Message (IM)
       5. By text message
       6. Other:______________________
  1. No, I will probably not tell this partner
  2. No, I will definitely not tell this partner

If D or E:

- - 1. What are some of the reasons why you do not plan to notify this partner (check all that apply):
       1. I am afraid that this partner will reject me
       2. I am afraid that this partner will become violent
       3. I am afraid that everyone in my community will find out about my infection
       4. I do not know how to contact this partner
       5. I do not think it is important to notify this partner
       6. Other:________________________
  1. I don’t know
  2. No answer

1. If there were an internet website available where you could send a free, anonymous email telling someone that they may have been exposed to an STI, how likely would you be to use it to notify this partner?
   1. I would definitely use it to notify this partner
   2. I would probably use it to notify this partner
   3. I would probably not use it to notify this partner
   4. I would definitely not use it to notify this partner

**Thinking about the next to last person you had sex with (Partner #2),**

1. Was this person (choose the best option):
   1. A stable or primary partner
   2. A casual or secondary partner
   3. An anonymous partner
   4. A sex work client
   5. A sex worker
   6. I don’t know
   7. No answer
2. Was this person:
   1. Male
   2. Female
   3. Transgender
   4. I don’t know
   5. No Answer
3. (For male partners) What do you think is this partner’s sexual identity?
   1. Heterosexual
   2. Bisexual
   3. Homosexual
   4. Transgender
   5. Other:_______________
   6. I don’t know
   7. No answer
4. (For male partners) What do you think is this partner’s sexual role?
5. Activo
6. Pasivo
7. Moderno
8. Other:______________
9. I don’t know

No answer

1. How many times have you had sex with this partner?
   1. 1 time
   2. 1-3 times
   3. 3-10 times
   4. More than 10 times
   5. I don’t know
   6. No answer
2. When was the last time you had sex with this partner?
   1. Less than one week ago
   2. Less than one month ago
   3. Less than three months ago
   4. Less than six months ago
   5. More than six months ago
   6. I don’t know
   7. No answer
3. Do you think you will have sex with this partner again in the future?
   1. I will definitely have sex with this partner again in the future
   2. I will probably have sex with this partner again in the future
   3. I will probably not have sex with this partner again in the future
   4. I will definitely not have sex with this partner again in the future
4. **During the past three months,** what sexual practices did you engage in with this partner?
5. You performed oral sex on them?

Y/N With a condom? Y/N

1. They performed oral sex on you?

Y/N With a condom? Y/N

1. They penetrated you?

Y/N With a condom? Y/N

1. You penetrated them (anal or vaginal intercourse)?

Y/N With a condom? Y/N

1. Other sexual acts? Y/N
   - 1. If so, please specify:___________________________________
2. I don’t know
3. No answer
4. How likely is it that you transmitted your STI to this partner?
5. Not at all likely or Impossible
6. Somewhat unlikely
7. Somewhat likely
8. Very likely or certain
9. I don’t know
10. No answer
11. How likely is it that this partner infected you (i.e., that they are the source of your STI)?
12. Not at all likely or Impossible
13. Somewhat unlikely
14. Somewhat likely
15. Very likely or certain
16. I don’t know
17. No answer
18. Do you know how to contact this partner?
19. In person (i.e., you know where they live or where they socialize)? Y/N
20. By telephone Y/N
21. By e-mail Y/N
22. By Instant Messenger (e.g., MSN Messenger, Yahoo Messenger, IM, etc.) Y/N
23. By another means? Y/N
    - 1. If so, please specify_________________________________________
24. I don’t know
25. No answer
26. Do you plan to tell (or have you already told) this partner about your diagnosis?
27. Yes, I have already told this partner
28. Yes, I will definitely tell this partner
29. Yes, I will probably tell this partner

If A, B, or C:

- - 1. What are some of the reasons why you plan to notify (or notified) this partner (check all that apply):
       1. To protect my partner’s health
       2. To protect my health
       3. To protect the health of men in my community
       4. To maintain trust in our relationship
       5. Other:______________________

1. What is the most likely way you would notify (or notified) this partner (check the best option)?
   - - 1. In person
       2. By telephone
       3. By e-mail
       4. By Instant Message (IM)
       5. By text message
       6. Other:______________________
2. No, I will probably not tell this partner
3. No, I will definitely not tell this partner

If D or E:

1. What are some of the reasons why you do not plan to notify this partner (check all that apply):
   - - 1. I am afraid that this partner will reject me
       2. I am afraid that this partner will become violent
       3. I am afraid that everyone in my community will find out about my infection
       4. I do not know how to contact this partner
       5. I do not think it is important to notify this partner
       6. Other:________________________
2. I don’t know
3. No answer
4. If there were an internet website available where you could send a free, anonymous email telling someone that they may have been exposed to an STI, how likely would you be to use it to notify this partner?
   1. I would definitely use it to notify this partner
   2. I would probably use it to notify this partner
   3. I would probably not use it to notify this partner
   4. I would definitely not use it to notify this partner

**Thinking about the third person you had sex with most recently (Partner #3),**

1. Was this person (choose the best option):
   1. A stable or primary partner
   2. A casual or secondary partner
   3. An anonymous partner
   4. A sex work client
   5. A sex worker
   6. I don’t know
   7. No answer
2. Was this person:
   1. Male
   2. Female
   3. Transgender
   4. I don’t know
   5. No Answer
3. (For male partners) What do you think is this partner’s sexual identity?
   1. Heterosexual
   2. Bisexual
   3. Homosexual
   4. Transgender
   5. Other:_______________
   6. I don’t know
   7. No answer
4. (For male partners) What do you think is this partner’s sexual role?
   1. Activo
   2. Pasivo
   3. Moderno
   4. Other:______________
   5. I don’t know
   6. No answer
5. How many times have you had sex with this partner?
   1. 1 time
   2. 1-3 times
   3. 3-10 times
   4. More than 10 times
   5. I don’t know
   6. No answer
6. When was the last time you had sex with this partner?
   1. Less than one week ago
   2. Less than one month ago
   3. Less than three months ago
   4. Less than six months ago
   5. More than six months ago
   6. I don’t know
   7. No answer
7. Do you think you will have sex with this partner again in the future?
   1. I will definitely have sex with this partner again in the future
   2. I will probably have sex with this partner again in the future
   3. I will probably not have sex with this partner again in the future
   4. I will definitely not have sex with this partner again in the future
8. **During the past three months,** what sexual practices did you engage in with this partner?
   1. You performed oral sex on them?

Y/N With a condom? Y/N

1. They performed oral sex on you?

Y/N With a condom? Y/N

1. They penetrated you?

Y/N With a condom? Y/N

1. You penetrated them (anal or vaginal intercourse)?

Y/N With a condom? Y/N

1. Other sexual acts? Y/N
   - 1. If so, please specify:___________________________________
2. I don’t know
3. No answer
4. How likely is it that you transmitted your STI to this partner?
5. Not at all likely or Impossible
6. Somewhat unlikely
7. Somewhat likely
8. Very likely or certain
9. I don’t know
10. No answer
11. How likely is it that this partner infected you (i.e., that they are the source of your STI)?
12. Not at all likely or Impossible
13. Somewhat unlikely
14. Somewhat likely
15. Very likely or certain
16. I don’t know
17. No answer
18. Do you know how to contact this partner?
19. In person (i.e., you know where they live or where they socialize)? Y/N
20. By telephone Y/N
21. By e-mail Y/N
22. By Instant Messenger (e.g., MSN Messenger, Yahoo Messenger, IM, etc.) Y/N
23. By another means? Y/N
    - 1. If so, please specify_________________________________________
24. I don’t know
25. No answer
26. Do you plan to tell (or have you already told) this partner about your diagnosis?
27. Yes, I have already told this partner
28. Yes, I will definitely tell this partner
29. Yes, I will probably tell this partner

If A, B, or C:

- - 1. What are some of the reasons why you plan to notify (or notified) this partner (check all that apply):
       1. To protect my partner’s health
       2. To protect my health
       3. To protect the health of men in my community
       4. To maintain trust in our relationship
       5. Other:______________________
    2. What is the most likely way you would notify (or notified) this partner (check the best option)?
       1. In person
       2. By telephone
       3. By e-mail
       4. By Instant Message (IM)
       5. By text message
       6. Other:______________________

1. No, I will probably not tell this partner
2. No, I will definitely not tell this partner

If D or E:

- - 1. What are some of the reasons why you do not plan to notify this partner (check all that apply):
       1. I am afraid that this partner will reject me
       2. I am afraid that this partner will become violent
       3. I am afraid that everyone in my community will find out about my infection
       4. I do not know how to contact this partner
       5. I do not think it is important to notify this partner
       6. Other:________________________

1. I don’t know
2. No answer
3. If there were an internet website available where you could send a free, anonymous email telling someone that they may have been exposed to an STI, how likely would you be to use it to notify this partner?
   1. I would definitely use it to notify this partner
   2. I would probably use it to notify this partner
   3. I would probably not use it to notify this partner
   4. I would definitely not use it to notify this partner

**Thank you for taking the time to answer our questions.**
