# Supplementary material for: Anticipated Notification of Sexual Partners following STD Diagnosis among Men Who Have Sex with Men and Transgender Women in Lima, Peru: A Mixed Methods Analysis
Source: PLoS One. 2016 Sep 29;11(9):e0163905. doi: 10.1371/journal.pone.0163905 (PMC5042523; doi:10.1371/journal.pone.0163905)
Supplement: S2 File — (DOCX) [file pone.0163905.s002.docx]

**S2 File. Interview Script. Qualitative semi-structured interview script.**

**PARTNER NOTIFICATION IN MSM**

**SUPPLEMENTARY INTERVIEW SCRIPT**

**(QUANTITATIVE STUDY; PHASE II)**

***Interviewer: Brief explanation of the project and verbal informed consent (attached).***

# Partner Notification

***Interviewer: We are going to discuss some issues related to partner notification after the diagnosis of a sexually transmitted infection (STI or STD). Please tell us only about your own attitudes, beliefts, and experiences, not those of your friends or partners.***

1. Have you ever heard of “partner notification”? (If so, elicit explanation).

***Yes, ‘Partner Notification’ means telling people you have recently had sex with that you have been diagnosed with an STI like gonorrhea, chlamydia, syphilis, genital herpes, or HIV.***

1. Do you think that it is important to notify recent sex partners if you have been diagnosed with an STI? (Elicit general perceptions of the importance of PN)
2. If someone you recently had sex with was diagnosed with an STI, would you want them to tell you? Would you expect them to tell you? (Elicit reasons why or why not)
   1. Are there certain types of partner or specific people in your life who you would think more or less likely to tell you? (Elicit different expectations based on partner type, length of relationship, etc.)
   2. Do you think that most of your friends would tell their partners if they were diagnosed with an STI?
   3. Do you think that most of the people in your neighborhood would tell their partners if they were diagnosed with an STI?
3. With regard to your own experience as someone diagnosed with HIV or syphilis, do you think you will try to notify all of your sex partners, some of your partners, or none of your partners?
   1. Are there specific people in your life, or certain types of partners, who you are more likely to notify? Less likely to notify? Why?
      1. Do you have someone who you consider a “stable” or “primary” partner? If so, do you think you are likely to notify this person? Why or why not?
      2. Do you have anyone who you consider “casual” or “secondary” partners? If so, do you think you are likely to notify these people? Why or why not?
      3. Do you have any partners who you have sex with in exchange for money or goods? If so, do you think you are likely to notify these people? Why or why not?
      4. Can you think of any other factors that are likely to influence your decisions about notification? For instance, do you think that your decisions about notification would be different for a male versus a female partner, for a gay versus a bisexual or heterosexual partner, for someone you had oral sex versus someone you had anal or vaginal sex with? Other factors?
4. What are some of the barriers that you anticipate could prevent you from notifying your partners? (Elicit external barriers including lack of contact information)
5. If it were available, would you be interested in using an internet partner notification system, where you could send an anonymous message to someone’s email address to tell them that you had been diagnosed with an STI?
   1. What do you think would be some of the advantages and disadvantages of such a system?
   2. If such a system were available, do you think it would make a difference in whether or not you notify your partners?
   3. Are there any specific features that you think it would be important to include in an internet notification system? (Potential prompts include educational information, links to testing and treatment resources, ability to send anonymous vs. named notification messages)

***Thank you for your participation. Is there anything else you would like to add about the issues that we have discussed? Are there any questions that I can answer for you?***
